# Emerging Strategies for the Bioremediation of the Phenylurea Herbicide Diuron

**DOI:** 10.3389/fmicb.2021.686509

**Published:** 2021-08-12

**Authors:** Jiayi Li, Wenping Zhang, Ziqiu Lin, Yaohua Huang, Pankaj Bhatt, Shaohua Chen

**Affiliations:** ^1^State Key Laboratory for Conservation and Utilization of Subtropical Agro-Bioresources, Guangdong Province Key Laboratory of Microbial Signals and Disease Control, Integrative Microbiology Research Centre, South China Agricultural University, Guangzhou, China; ^2^Guangdong Laboratory for Lingnan Modern Agriculture, Guangzhou, China

**Keywords:** diuron, ecotoxicity, bioremediation, metabolic pathways, molecular mechanisms, biodegradation

## Abstract

Diuron (DUR) is a phenylurea herbicide widely used for the effective control of most annual and perennial weeds in farming areas. The extensive use of DUR has led to its widespread presence in soil, sediment, and aquatic environments, which poses a threat to non-target crops, animals, humans, and ecosystems. Therefore, the removal of DUR from contaminated environments has been a hot topic for researchers in recent decades. Bioremediation seldom leaves harmful intermediate metabolites and is emerging as the most effective and eco-friendly strategy for removing DUR from the environment. Microorganisms, such as bacteria, fungi, and actinomycetes, can use DUR as their sole source of carbon. Some of them have been isolated, including organisms from the bacterial genera *Arthrobacter*, *Bacillus*, *Vagococcus*, *Burkholderia*, *Micrococcus*, *Stenotrophomonas*, and *Pseudomonas* and fungal genera *Aspergillus*, *Pycnoporus*, *Pluteus*, *Trametes*, *Neurospora*, *Cunninghamella*, and *Mortierella.* A number of studies have investigated the toxicity and fate of DUR, its degradation pathways and metabolites, and DUR-degrading hydrolases and related genes. However, few reviews have focused on the microbial degradation and biochemical mechanisms of DUR. The common microbial degradation pathway for DUR is *via* transformation to 3,4-dichloroaniline, which is then metabolized through two different metabolic pathways: dehalogenation and hydroxylation, the products of which are further degraded *via* cooperative metabolism. Microbial degradation hydrolases, including PuhA, PuhB, LibA, HylA, Phh, Mhh, and LahB, provide new knowledge about the underlying pathways governing DUR metabolism. The present review summarizes the state-of-the-art knowledge regarding (1) the environmental occurrence and toxicity of DUR, (2) newly isolated and identified DUR-degrading microbes and their enzymes/genes, and (3) the bioremediation of DUR in soil and water environments. This review further updates the recent knowledge on bioremediation strategies with a focus on the metabolic pathways and molecular mechanisms involved in the bioremediation of DUR.

## Introduction

Diuron [1-(3,4 dichlorophenyl)-3,3 dimethyl urea] (DUR) is an active ingredient in the formulation of several plant protection products and biocides. It is widely used for the pre- or postemergence control of various types of broadleaf and grassy weeds in diverse crops such as cotton, fruit, and cereals, as well as for algal control in fish production ponds, with application doses ranging from 0.45 to 3 kg a.i. ha^–1^ (Castillo et al., 2006; Stork et al., 2008; Lu et al., 2019; Tandon and Pant, 2019). It has also been used in antifouling paints for boating activities and non-crop applications such as roads, garden paths, and railway lines (Schrader et al., 2004; Hussain et al., 2015).

Continuous use of DUR has resulted in the contamination of the environment and raised public concern about its impact on human health. In mammals, including humans, DUR (0.05–0.5 μg/L) is suspected of having carcinogenic, mutagenic, and neurotoxic effects, causing genotoxicity, cytotoxicity, embryotoxicity, and immunotoxicity, as well as a disruption of endocrine, respiratory, and cardiovascular processes (da Rocha et al., 2013; Behrens et al., 2016; Manonmani et al., 2020). This compound (200 ng/L) is also harmful to fish, plants, aquatic invertebrates, freshwater algae, and microbial species (Pesce et al., 2010; Pereira et al., 2015; Wilkinson et al., 2017; Pei et al., 2020). In addition, some metabolites of DUR, such as 3,4-dichloroaniline (3,4-DCA) and 3-(3,4-dichlorophenyl)-1-methylurea (DCPMU), showed more ecotoxicological effects than their parent compound (Stork et al., 2008; Hussain et al., 2015). Therefore, remediation strategies *in situ* should decrease their persistence, avoid their transfer, and have a positive impact on terrestrial and living organisms.

Various technologies have emerged to remove DUR from the environment and reduce its harmful effects, including advanced physical adsorption, photocatalytic degradation, chemical degradation, and biological treatments (da Silva Teófilo et al., 2020; de Souza and Dos Santos, 2020; Park and Jhung, 2020; Silambarasan et al., 2020). However, previous studies have reported that the by-products of the abiotic transformation of this compound are more hazardous than the parent compound itself (Liu et al., 2018; Manonmani et al., 2020). Moreover, DUR can be released back into the soil after the formation of bound residues *via* physical adsorption (Dias Guimaraes et al., 2018). Microbial degradation has advantages over other degradation methods because it is cost-effective and environmentally friendly, making it well suited for the bioremediation of many organic pollutants from different environments (Peng et al., 2012; Birolli et al., 2019; Bhatt et al., 2021b). Numerous bacteria, fungi, and actinomycetes have been isolated and identified as capable of degrading DUR (Ellegaard-Jensen et al., 2014; Hussain et al., 2015; Grandclement et al., 2017; Villaverde et al., 2017, 2018). However, few reviews have focused on the microbial degradation and biochemical mechanisms of DUR (Giacomazzi and Cochet, 2004; Hussain et al., 2015).

The present review summarizes up-to-date information on DUR in its environmental occurrence and toxicity, along with the newly isolated and characterized DUR-degrading microorganisms and their application for the bioremediation of DUR in soil and water environments. This review emphasizes the metabolic pathways and degradation mechanisms that dissipate DUR, with additional focus on the advances of hydrolases and related genes, to provide novel facts underlying the metabolic pathways governing these processes. Finally, environmental factors affecting the practical application of microorganisms for the bioremediation of DUR are explained, providing a better understanding of how microorganisms promote the natural dissipation of DUR, thereby limiting its dispersion in the environment.

## Environmental Occurrence and Toxicity

### Environmental Occurrence

**FIGURE 1:**
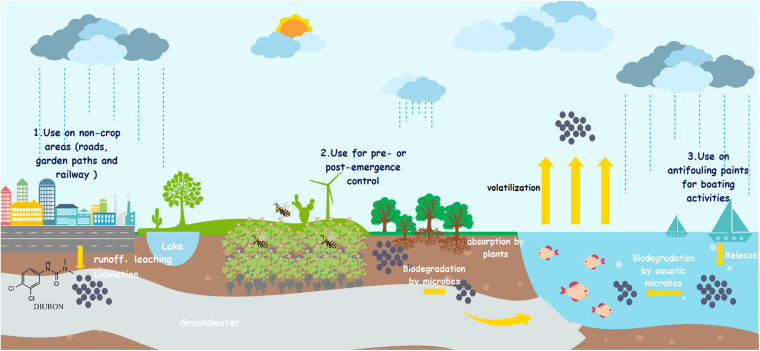
Fate and occurrence of diuron into the environment. (1) Diuron enters groundwater through leaching. (2) Diuron enters surface water through runoffs. (3) Diuron enters atmosphere through volatilization.

Due to their high persistence and extensive use, DUR residues are frequently found in water, soil, and sediments (Field et al., 2003; Giacomazzi and Cochet, 2004; Tandon and Pant, 2019). The highest concentration of DUR in sediment samples in the Brazilian Amazon region was 55.2 μg/kg (Viana et al., 2019). In Costa Rica river basins, the highest DUR concentration in water and sediment samples were 22.8 μg/L and 11.75 μg/kg, respectively (Carazo-Rojas et al., 2018). Loos et al. (2009) reported that approximately 70% of samples from European streams contained the maximum concentration of DUR at 864 ng/L. In Europe, the maximum allowable concentration and annual average concentration of DUR in surface water are set to 1.8 and 0.2 μg/L, respectively, with DUR already being included in the 2019 European Commission Priority Substance list (Mori et al., 2018). The European Union guideline value for DUR in drinking water is 0.1 μg/L (DWI, 2013). In addition to the parent compound, DUR degradation metabolites have also been detected in soil and aquatic environments around the world (Hussain et al., 2015). Moreover, its relatively high solubility and long aqueous photolysis half-life (DT_50_) make it available in water fractions such as rivers, streams, lakes, and seawater (Felício et al., 2018). The European Food Safety Authority reported that the DT_50_ of DUR in the soil ranges from 14 to 372 days under aerobic conditions (EFSA, 2005). Mercurio et al. (2016) reported that the DT_50_ of DUR in water was 499 days in a dark environment, suggesting very slow natural degradation rates. Several reports have confirmed that DUR enters the surface water and groundwater through irrigation, drainage, percolation, and surface runoff (Camenzuli et al., 2012; Moisset et al., 2015; Maqbool et al., 2016), as shown in Figure 1.

**FIGURE 2:**
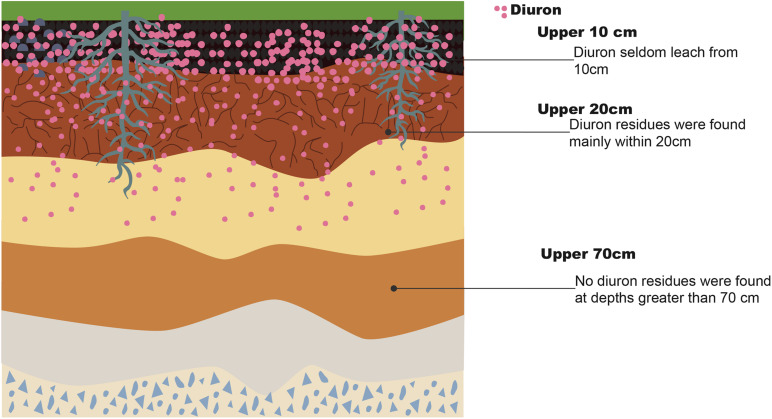
Distribution of diuron in upper soil.

The residues of DUR are mainly distributed within the most superficial layer of the topsoil, seldom leaching from the upper 10 cm of surface soil, with no residues detected at depths greater than 70 cm. DUR concentration tends to decline with soil depth (Tworkoski et al., 2000; Landry et al., 2006), as shown in Figure 2. The sorption of non-ionic organic compounds in the soil is usually expressed as the sorption coefficient (*K*_D_), reflecting the dissipation dynamics of xenobiotics in the environment. Dages et al. (2015) reported that the *K*_D_ of DUR decreased from 13.93 mL/g to 3.57 mL/g from the upper soil (0–17 cm) to deeper soil (45–80 cm); a similar value was reported by a previous study (Dores et al., 2009). DUR is considered weakly to moderately mobile, being strongly retained in the topsoil but less retained in deeper regions. Dos Reis et al. (2017) reported that 96.1% of DUR remained in the 0–5-cm soil layer after a rainfall simulation, whereas only 0.91% remained in the 5–10-cm soil layer, confirming its low mobility through the soil profile. DUR mobility is related to soil organic carbon content and soil texture (Landry et al., 2006). Tandon and Pant (2019) found that the persistence of DUR was stronger in sandy loam than in silty clay loam soil in sugarcane fields. The DT_50_ of DUR in silty clay loam soil was 32.37 days when applied at 4 kg/ha, whereas it was 43.93 days in sandy loam soil. Dias Guimaraes et al. (2018) found that among the three types of soil, namely, sand, clay, and loam, which had been exposed to herbicides for the past 3 years, the DT_50_ of loam soil was the lowest (97.63 days). Loam soil showed the highest cation exchange capacity among the three types of soil; therefore, the benefit to growth activity and life of microorganisms due to the availability of more nutrients increases DUR degradation. When herbicides are converted to bound residues (non-extractable residues), they become unavailable for biodegradation by microorganisms. They also determined that the bound residues of DUR accounted for 38.65–42.54% of the initial amount (3.32 a.i. g^–1^ of soil) at 70 days after treatment. The DUR metabolites DCPMU and 3,4-dichlorophenylurea (DCPU) were also found in the soil. This is possibly due to its high microbial activity, as the 0–2.5-cm surface soil with grass cover contained a large amount of DCPMU, where DCPMU migrated to a depth of 10–20 cm in the soil (Landry et al., 2006). DCPMU was detected at a depth of 40 cm in natural calcareous soils (Field et al., 2003).

Diuron is frequently detected in rivers, streams, lakes, and seawater because of its high water solubility (42 mg/L) and long aqueous photolysis half-life (Felício et al., 2018; Akcha et al., 2020). Loos et al. (2009) reported that approximately 70% of the samples from European streams contained the maximum concentration of DUR (864 ng/L). In the Mediterranean and in Australia, stream waters and suspended sediments transport pesticides from agricultural areas to coastal lagoons and wetlands, leading to the accumulation of active ingredients in the sediments (Moreno-González and León, 2017; Mendez et al., 2018). Landry et al. (2006) showed that the vineyards of Burgundy were contaminated with DUR residues. They also detected greater quantities of DCPMU than DCPUs in the percolates, especially after rainfall. In Costa Rica river basins, the highest DUR concentrations in water and sediment samples were 22.8 μg/L and 11.75 μg/kg, respectively. Many studies have indicated that the risk is unacceptable even under a conservative scenario and suggested a high degree of acute toxicity to the ecosystem (Carazo-Rojas et al., 2018; Viana et al., 2019). In the Galápagos Islands, one of the last frontiers for conducting ecotoxicology research (UNESCO World Heritage region), the concentration of DUR (1.61 μg/L) between the two islands was the highest in samples from coastal waters in urban areas. Riascos-Flores et al. (2020) showed that this concentration (1.61 μg/L) poses a high risk for three groups of organisms: algae, invertebrates, and fishes.

### Direct and Indirect Effect on Aquatic Organisms, Mammals, and Humans

Numerous non-target organisms, such as bacteria, phytoplankton, invertebrates, fish, mammals, and humans, suffer from the deleterious effects of xenobiotics (Giacomazzi and Cochet, 2004; Dupraz et al., 2016). As photosystem II and its conservation in plants are the targets of DUR, non-target phytoplankton is equally affected, such as seagrass and corals (Marques et al., 2020; Thomas et al., 2020). Plants eventually die due to long-term starvation under moderate irradiation (electron transfer rate inhibition) or oxidative stress under higher levels of radiation (Diepens et al., 2017; Wilkinson et al., 2017). Over the past decade, a large volume of published studies has described the toxic effects of DUR on green algae and seagrass in tropical marine environments (King et al., 2013; Negri et al., 2015; Diepens et al., 2017; Brodie and Landos, 2019; Thomas et al., 2020). Negri et al. (2015) reported 7-day IC_50_ values (concentrations inhibiting quantum yield by 50%) of 2.7 and 3.8 μg/L for DUR on the growth of two tropical seagrass species, *Zostera muelleri* and *Halodule uninervis*, respectively. Moreover, phytoplankton could suffer from an additive toxicity effect with other adverse factors, such as ocean warming and ocean acidification (Marques et al., 2020). van Dam et al. (2015) conducted co-exposure experiments, revealing that the inhibition of photosynthetic yield under DUR and thermal stress is additive. In addition, the ocean contains other pesticide-active ingredients that can also cause additional toxic effects on phytoplankton. Mercurio et al. (2018) evaluated the potential toxicity of photosystem II herbicide metabolites to coral symbionts (*Symbiodinium* sp.) and found that the toxicity of 3,4-DCA was greater than that of DUR. Likewise, Sigurnjak et al. (2020) revealed additive toxicity effects on freeze-dried *Vibrio fischeri* when DUR was combined with alachlor, chlorfenvinphos, and isoproturon.

Recently, investigators have examined the effects of DUR on fish and invertebrates. For example, Akcha et al. (2020) showed the genotoxicity of DUR through DNA damage and a decrease in DNA methylation levels in oysters. Boscolo et al. (2018) showed that the biotransformation of DUR to its active metabolites affects neurotransmitters in Nile tilapia. Similarly, Perissini-Lopes et al. (2016) reported that the DUR metabolites, 3,4-DCA, DCPU, and DCPMU, have antiandrogen activity in Nile tilapia, potentially causing reproductive disorders in male fish.

Indeed, it is evident that phenylurea herbicides (PUHs) have relatively low acute toxicity to mammals, birds, and fish compared with plants, algae, and invertebrates. However, at sufficiently high concentrations, DUR was shown to have toxic effects on fetal development in mammals, birds, and humans (Huovinen et al., 2015; Eletto et al., 2020). Several studies have demonstrated that DUR is carcinogenic to the rat urothelium (Fernandes et al., 2007; da Rocha et al., 2013; Ihlaseh-Catalano et al., 2014). Huovinen et al. (2015) and Eletto et al. (2020) found that it had cytotoxic effects on human primary urothelial cells at high concentrations and elucidated the molecular mechanisms involved in this process. Mohammed et al. (2018) detected that DUR can penetrate the human placenta and metabolize it to DCPMU at a high concentration (100 μM). This suggests that pregnant women can suffer fetotoxicity if they are exposed to DUR. Mohammed et al. (2020) indicated that DUR metabolites are more toxic than the parent compound in human cells, with the mitochondria as their target. Concerning the effects of chronic exposure to herbicides and the mechanisms involved, several questions regarding ecotoxicology remain to be addressed (Akcha et al., 2020).

## Diuron-Degrading Microorganisms

### Axenic Cultures for Diuron Degradation

In recent years, the use of biological resources, especially microbes, to degrade DUR has emerged as a powerful tool for its degradation and remediation *in situ* (Bhatt et al., 2021a,c; Zhang et al., 2021). DUR is susceptible to microbial degradation, and aquatic microorganisms enriched in pond water can also degrade DUR to one of its major metabolites, 3,4-DCA (Richards et al., 2020). The first known bacterial strain capable of mineralizing DUR, *Variovorax* sp. SRS16, was isolated by Sorensen et al. (2008). This bacterial strain was also the first bacterium capable of mineralizing both the *N*,*N*-dimethyl-substituted PUH, linuron, and the *N*-methoxy-*N*-methyl-substituted PUH, DUR. Currently, a number of DUR-degrading bacteria and fungi have been screened, enriched, and cultivated, including the organisms from the genera *Pseudomonas*, *Stenotrophomonas*, *Arthrobacter*, *Burkholderia*, *Vagococcus*, *Bacillus*, and *Micrococcus* (Table 1) and the fungal genera *Pycnoporus*, *Pluteus*, *Trametes*, *Neurospora*, *Cunninghamella*, *Aspergillus*, and *Mortierella* (Table 2). To date, only a few bacterial strains capable of mineralizing DUR or simultaneously degrading DUR and 3,4-DCA have been reported (Sharma et al., 2010; Ellegaard-Jensen et al., 2013; Villaverde et al., 2017), as most isolates catabolize DUR to DCPMU, DCMU, or 3,4-DCA. *Arthrobacter* sp. BS1, BS2, and SED1 can transform DUR to 3,4-DCA, whereas *Achromobacter* sp. SP1 was able to degrade 3,4-DCA through cooperative microbial transformation. An artificial consortium comprising BS2 and SP1 was able to completely mineralize DUR within 5 days (Devers-Lamrani et al., 2014).

**TABLE 1:** Newly isolated diuron-degrading bacteria.

**Strains**	**Findings**	**Intermediates**	**Sources**	**References**
*Micrococcus* sp. PS-1	96% of diuron (250 mg/L) was degraded in 30 h	3,4-DCA, 1,2-DCB, 4,5-DCC, 3,4-DCHD, 3-COHDA	Soil from diuron storage site, Ankleshwar, India	Sharma et al., 2010
*Bacillus cereus*, *Vagococcus fluvialis*, *Burkholderia ambifaria*, *Bacillus* spp.	21, 25, 22, and 19%, of diuron (40 mg/L) was degraded in 35 days, respectively	DCPMU, 3,4-DCA	Sugarcane-cultivated fields, Kenya	Ngigi et al., 2011
*Arthrobacter* sp. BS2	100% of diuron (30 mg/L) was degraded in 24 h	3,4-DCA	Soil and sediments, France	Devers-Lamrani et al., 2014
*Arthrobacter* sp. BS1, SED1	100% of diuron (50 mg/L) was degraded in 5 days	3,4-DCA	Soil and sediments, France	Devers-Lamrani et al., 2014
*Bacillus licheniformis* SDS12	85.6% of diuron (50 mg/L) was degraded in 10 days	3,4-DCA, 1,2-DCB, 4,5-DCC, 3,4-DCHD, 3-COHDA	*Parthenium* endophyte, India	Singh and Singla, 2019
*Stenotrophomonas rhizophila* CASB3	94% of diuron (50 mg/L) was degraded in 42 days	3,4-DCA, 4-CA, aniline, catechol	Endophyte from roots of *Fragaria ananassa* plants, Chile	Silambarasan et al., 2020
*Pseudomonas aeruginosa* FN	54% of diuron (10 mg/L) was degraded in 6 h	3,4-DCA	Tobacco waste, Croatia	Grgić et al., 2020
*Bacillus pseudomycoides* D/T, *Bacillus simplex/Bacillus muralis* D/N	54 and 51% of diuron (50 mg/L) was degraded in 46 days, respectively	DCPMU, 3,4-DCA	Diuron contaminated sugarcane and pineapple-cultivated soils, Kenya	Muendo et al., 2021

*3,4-DCA, 3,4-dichloroaniline; DCPMU, 1-(3,4-dichlorophenyl)-3-methylurea; 4-CA, 4-chloroaniline; 3-COHDA, 3-chloro-4-oxohexanedioic acid; 3,4-DCHD, 3,4-dichlorohex-3-ene-1,6-diol; 4,5-DCC, 4.5-dichlorocatechol; 1,2-DCB, 1,2-dichlorobenzene; DCPU, 1-(3,4-Dichlorophenyl) urea.*

**TABLE 2:** Newly isolated diuron-degrading fungi.

**Strains**	**Findings**	**Intermediates**	**Sources**	**References**
*Mortierella* sp. LEJ701 and LEJ702	100, 33.6% of diuron (5 mg/L) was degraded in 43 days, respectively	DCPMU, DCPU, DCPMDU	Agricultural field, Denmark	Ellegaard-Jensen et al., 2013
*Aspergillus brasiliensis* G08, *Aspergillus* sp. G25, and *Cunninghamella elegans* B06	81.3, 84.6, and 67.0% of diuron (10 mg/L) was degraded in 5 days	DCPMU, DCPU, 3,4-DCA	Sugarcane-cultivated fields, Brazil	Egea et al., 2014; Perissini-Lopes et al., 2016
*Neurospora intermedia* DP8-1	99% of diuron (50 mg/L) was degraded in 3 days	DCPMU, DCPU	Endophyte from sugarcane root, China	Wang et al., 2017
*Trametes versicolor* K-41	98.7% of diuron (1 μM) was degraded in 14 days	DCPMU, DCPU, 3,4-DCA	Natural decayed wood, Japan,	Mori et al., 2018
*Pluteus cubensis* SXS 320	96.8% of diuron (25 mg/L) was degraded in 20 days	DCPMU, DCPU, 3,4-DCA	Decaying wood, Brazil	Henn et al., 2020
*Pycnoporus sanguineus* MCA 16	56% of diuron (25 mg/L) was degraded in 40 days	DCPMU, DCPU, 3,4-DCA	Decaying wood, Brazil	Henn et al., 2020

*DCPMU, 1-(3,4-dichlorophenyl)-3-methylurea; DCPU, 1-(3,4-dichlorophenyl) urea; DCPMDU, 1-(3,4-dichlorophenyl)-3-methylideneurea; 3,4-DCA, 3,4-dichloroaniline.*

Enrichment culture is an important step for scanning DUR degraders, as most degrading microorganisms have been isolated from the soil, sediment, and agricultural fields (Ellegaard-Jensen et al., 2014; Villaverde et al., 2017, 2018). In addition, isolation from plant rhizosphere, roots, or decaying plant tissues is being increasingly recognized as an important source of DUR-degrading microorganisms (Bhatt et al., 2020; Huang et al., 2020; Pang et al., 2020). For example, some endophytes capable of degrading DUR have been isolated from plant roots (Wang et al., 2017; Grgić et al., 2020), which may have evolved mechanisms to degrade toxic pollutants into benign forms (Singh and Singla, 2019). For example, Wang et al. (2017) isolated an endophyte, *Neurospora intermedia* strain DP8-1, from sugarcane. The removal rate of this endophyte for DUR in an aqueous medium was 99% within 3 days; in sterile soil, it was 41.92% within 20 days, and it was mainly metabolized to DCPMU and DCPU.

Moreover, plant growth-promoting rhizobacteria have played a positive role in the degradation of herbicides in agricultural soils. Silambarasan et al. (2020) isolated *Stenotrophomonas rhizophila* strain CASB3, which reached a degradation rate of 94% within 42 days from an initial concentration of 50 mg/L DUR, and simultaneously enhanced the root–shoot length, fresh–dry biomass, and photosynthetic pigments in *Lactuca sativa* plants, which also showed their ability to degrade DUR under saline stress conditions. Similarly, Singh and Singla (2019) isolated five DUR-degrading endophyte strains with PGP traits. The degradation rate of 50 mg/L DUR by *Bacillus licheniformis* strain SDS12 was 85.6% within 10 days.

Fungi have also been widely used in the biodegradation and bioremediation of DUR. White-rot fungi have great potential for degrading DUR due to their high levels of enzyme activity, including extracellular ligninolytic enzymes, intracellular cytochrome P450 monooxygenases, and antioxidant enzymes (Coelho-Moreira et al., 2018; Mori et al., 2018; Hu et al., 2020). Some white-rot fungi degrade DUR to DCPMU and DCPU and do not accumulate the metabolite 3,4-DCA (Ellegaard-Jensen et al., 2013; Coelho-Moreira et al., 2018). Mori et al. (2018) found that the white-rot fungus *Trametes versicolor* K-41 could utilize 3,4-DCA as its sole carbon and energy source in an aqueous medium. There may be a mechanism involved in the rapid degradation of 3,4-DCA, protecting fungi from high toxicity, or there might be a new metabolic pathway involved in this phenomenon.

### Consortium-Based Removal of Diuron

The isolation and purification of microorganisms capable of degrading PUHs have often failed, partly because of the requirement of catabolic cooperation between microbial populations of bacterial consortia (Sørensen et al., 2002; Bhatt et al., 2021d; Huang et al., 2021). In addition, complementary catabolism between synergistic species in a consortium seldom leaves harmful intermediates (Zhang et al., 2018, 2020). *Sphingomonas* sp. SRS2 is auxotrophic, meaning it requires nutrition from other microorganisms. Therefore, strain SRS2 uses the amino acids supplied by strain SRS1, leading to the corresponding growth of strain SRS2 and the rapid metabolism of isoproturon to carbon dioxide (CO_2_) (Sørensen et al., 2002). Villaverde et al. (2017) found that a consortium composed of three DUR degraders, *Arthrobacter sulphonivorans*, *Variovorax soli*, and *Advenella* sp. JRO completed DUR mineralization after only a few days. When combined in pairs, they mineralized 40% of DUR in solution; however, none of the three strains individually mineralized DUR. By consuming excretion products from *Comamonas testosteroni* WDL7 and/or *Hyphomicrobium sulfonivorans* WDL6, *Variovorax* sp. WDL1 can indirectly obtain carbon and nutrients from linuron (Albers et al., 2018). They observed that the linuron hydrolase gene *hylA* in WDL1 was expressed over 100-fold higher in a consortium condition. Zhang et al. (2018) isolated a consortium that achieved complete metabolism of DUR. *Diaphorobacter* sp. strain LR2014-1 initially degraded linuron to 3,4-DCA, whereas *Achromobacter* sp. strain ANB-1 further mineralized 3,4-DCA to CO_2_. In addition to the original syntrophic consortia described earlier, a considerable number of recent studies have used artificially composed consortia to degrade PUHs. Sorensen et al. (2008) conducted an experiment using a linuron degrader (*Variovorax* sp. SRS16) and a DUR degrader (*Arthrobacter globiformis* D47) and found that neither of them individually mineralized DUR in a liquid medium, but when combined, the consortium mineralized 31–62% of DUR to CO_2_. A consortium composed of bacteria (*Sphingomonas* sp., *Variovorax* sp., and *A. globiformis*) and fungi (*Mortierella* sp. LEJ702 and LEJ703) achieved complete mineralization of DUR in sand (Ellegaard-Jensen et al., 2014). The DUR degrader *Arthrobacter* sp. BS2 and 3,4-DCA degrader *Achromobacter* sp. SP1 were artificially combined and were able to entirely dissipate DUR within 35 h (Devers-Lamrani et al., 2014). On the other hand, Dejonghe et al. (2003) showed that the degradation rate of linuron by an artificial consortium was lower than that of the original consortium, possibly because species had a lower abundance in the artificial consortium. According to Zhang et al. (2018), functional species are not always the predominant species in a community. Strains LR2014-1 (0.33%) and ANB-1 (0.77%) can hydrolyze linuron to 3,4-DCA and mineralize 3,4-DCA but only at a low abundance in a consortium.

## Microbial Degradation Pathways and Mechanisms

**FIGURE 3:**
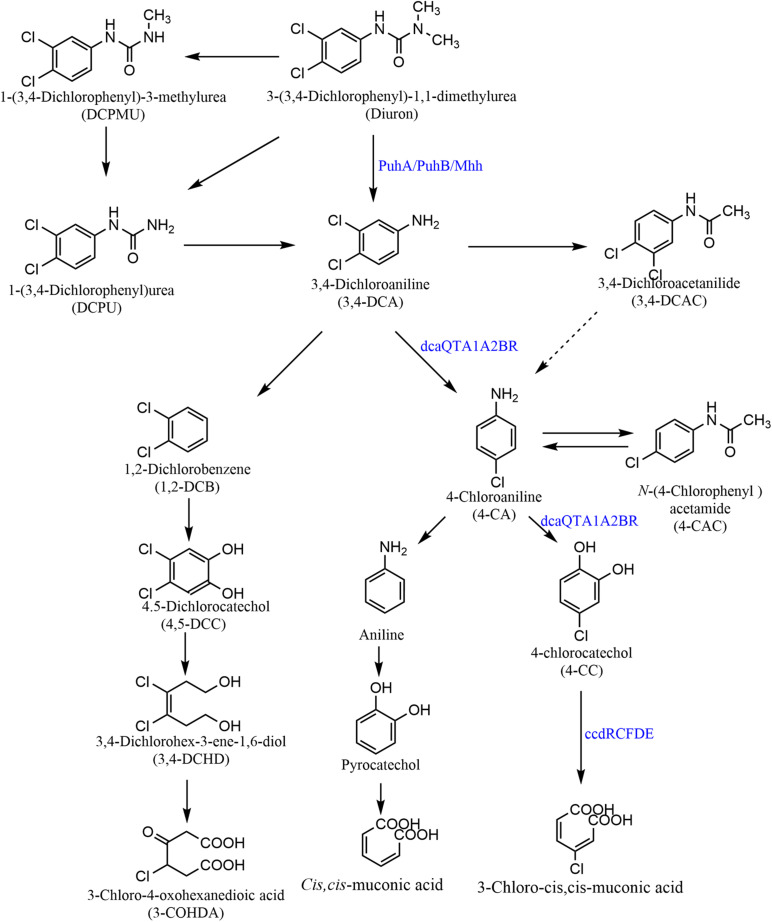
Microbial degradation pathways of diuron. Reported diuron hydrolases convert diuron to 3,4-DCA, including PuhA, PuhB, and Mhh (Khurana et al., 2009; Zhang et al., 2018). A multicomponent dioxygenase complex encoded by *dcaQTA1A2BR* further mineralizes 3,4-DCA to 4-chlorocatechol. Metabolite 4-chlorocatechol is further degraded through a modified ortho-cleavage pathway encoded by *ccdRCFDE* (Bers et al., 2013).

Microbial metabolism accounts for a large proportion of the degradation and natural attenuation of DUR in the environment. The degradation pathways of DUR in microorganisms are summarized in Figure 3. First, DUR degrades into one or two *N*-demethylations of a urea group and generates two metabolites, DCPMU and DCPU, respectively. This is followed by the hydrolysis of the amide bond, generating the metabolite 3,4-DCA, which is the common microbial DUR degradation product (Giacomazzi and Cochet, 2004; Egea et al., 2017; Silambarasan et al., 2020). Studies have also shown that some strains directly transform DUR to DCPU or 3,4-DCA without the formation of other intermediates (Cui et al., 2014; Hussain et al., 2015). Hydrolysis of amide bonds to produce 3,4-DCA is a key enzymatic step in the degradation of DUR (Hussain et al., 2015). According to the current literature, 3,4-DCA degradation mainly proceeds through two different metabolic pathways: dehalogenation and hydroxylation. The first biodegradation pathway of 3,4-DCA proceeds *via* direct oxidative deamination to form 1,2-dichlorobenzene and subsequent ortho-cleavage of the resulting 4,5-dichlorocatechol, followed by the phenyl ring breakage of 4,5-dichlorocatechol to produce 3,4-dichlorohex-3-ene-1,6-diol and 3-chloro-4-oxohexanedioic acid. 3-Chloro-4-oxohexanedioic acid subsequently enters the succinic acid degradation pathway, which has been reported by Sharma et al. (2010) and Singh and Singla (2019). The second biodegradation pathway of 3,4-DCA is *via* dechlorination from the aromatic ring and the generation of the mono-chlorinated aniline, 4-chloroaniline (4-CA) (Silambarasan et al., 2020). Degradation of 4-CA also proceeds through two different pathways: dechlorination and deoxygenation, forming aniline and 4-chlorocatechol, respectively. According to Hongsawat and Vangnai (2011), *Acinetobacter baylyi* GFJ2 can catabolize 4-CA through both these pathways, and after that, aniline deamination, to generate catechol. Then, ortho-cleavage of the phenyl ring leads to the accumulation of *cis*,*cis-*muconic acid and 3-chloro-*cis*,*cis*-muconic acid, respectively, in the pyrocatechol and 4-chlorocatechol pathways. However, Egea et al. (2017) and Silambarasan et al. (2020) showed that catechol is the final metabolite after the deamination of aniline. Ellegaard-Jensen et al. (2013, 2014) and Perissini-Lopes et al. (2016) found that some fungi, including *Mortierella isabellina*, *Aspergillus brasiliensis* G08, *Cunninghamella elegans* B06, *Mortierella* sp. LEJ702, and *A. globiformis* D47, metabolize 3,4-DCA to 3,4-dichloroacetanilide (3,4-DCAA). These results are similar to those reported by Tixier et al. (2002). Although this process does not achieve complete mineralization, toxicity is reduced, as 3,4-DCAA is less toxic than 3,4-DCA (Tixier et al., 2002). According to Egea et al. (2017), some isolates act on 3,4-DCA and 4-CA to form 3,4-DCAA and *N*-(4-chlorophenyl) acetamide *via* alkylation and dealkylation. However, *Micrococcus luteus* and *Achromobacter* sp. act on 3,4-DCA and dehalogenate it directly to aniline. DUR is metabolized *via* a different pathway under anaerobic conditions, and Attaway et al. (1982) detected the accumulation of 3-(3-chlorophenyl)-1-1dimethylurea through this mechanism. However, Stasinakis et al. (2009) indicated that DCPU is the major metabolite under anoxic conditions. Three intermediate metabolites, DCPMU, DCPU, and DCA, were detected under aerobic conditions, but only DCPU was detected under anoxic conditions, which exhibited a longer half-life (92 *vs*. 35 days under aerobic conditions) (Shareef et al., 2014). However, there is little published information on the DUR degradation pathway under anaerobic conditions.

Although the DUR degradation pathway has been partly elucidated, the complete metabolic pathway and the genes and enzymes involved in metabolism remain to be discovered. Advanced omics-based approaches will serve as the baseline information for genetic engineering leading to DUR degradation. The sequence information obtained through these approaches could be used to identify potential hosts and study gene exchanges in bacterial populations. The mechanism involved in these strains also requires further investigation.

## Functional Enzymes and Genes Involved in Diuron Degradation

**FIGURE 4:**
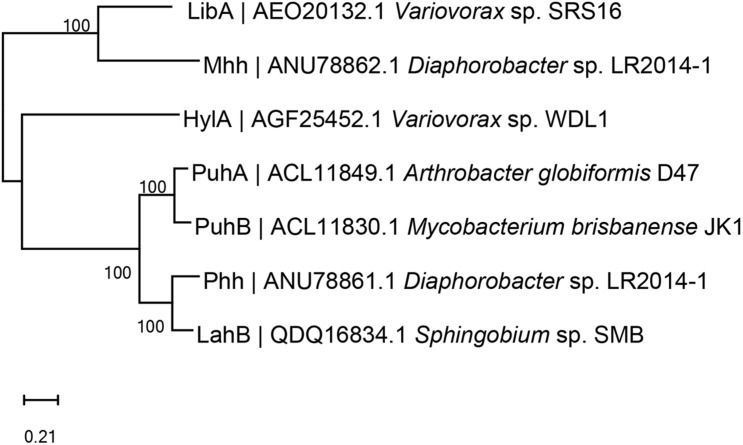
Phylogenetic tree of the key phenylurea herbicide initial hydrolysis enzymes constructed *via* neighbor-joining method (amino acid sequences). Code before strain name is National Center for Biotechnology Information accession number. PuhA was isolated from *Arthrobacter globiformis* D47 (Khurana et al., 2009). PuhB was isolated from *Mycobacterium brisbanense* JK1 (Khurana et al., 2009). LibA was isolated from *Variovorax* sp. SRS16 (Sørensen et al., 2005). HylA was isolated from *Variovorax* sp. WDL1 (Dejonghe et al., 2003). Phh and Mhh were isolated from *Diaphorobacter* sp. LR2014-1 (Zhang et al., 2018). LahB was isolated from *Sphingobium* sp. SMB (Zhang et al., 2020).

Many different enzymatic processes are involved in biodegradation by microorganisms, including hydroxylation, demethylation, dechlorination, and oxidation (Liu et al., 2020; Bhatt et al., 2021e). Different biodegradation processes are associated with different enzymes, such as hydrolases, esterases, dehydrogenases, laccases, and lignin peroxidases (Maqbool et al., 2016; Lin et al., 2020; Mishra et al., 2020). The reported enzymes involved in the initial degradation of PUHs are amidohydrolases or amidases, which are summarized in Table 3, and a phylogenetic tree (amino acid sequences) of the key initial hydrolytic enzymes is shown in Figure 4.

**TABLE 3:** Phenylurea herbicide initial hydrolysis enzymes.

**Enzymes**	**Type**	**GenBank accession No.**	**Degraded herbicides**	**Biochemical conditions**	**Host strains**	**Host strain sources**	**References**
PuhA	Metal-dependent hydrolase subfamily, amidohydrolase superfamily	ACL11849.1	DM and OM	pH 6.5–8.5, Temp 30–35°C, *K*_m_ 55.0 ± 19.1 μM (for diuron, different *K*_m_ calculated for all the substrates), MW 48.9 KDa, pI 5.2	*Arthrobacter globiformis* D47	Isoproturon-treated cereal-growing areas, United Kingdom	Cullington and Walker, 1999; Turnbull et al., 2001; Khurana et al., 2009
PuhB	Metal-dependent hydrolase subfamily, amidohydrolase superfamily	ACL11830.1	DM and OM	pH 6.5–8.5, Temp 30–35°C, *K*_m_ 11.0 ± 2.8 μM (for diuron, different *K*_m_ calculated for all the substrates), MW 49.5 KDa	*Mycobacterium brisbanense* JK1	Diuron-treated sugarcane-growing areas, Australia	Khurana et al., 2009
LibA	Amidase superfamily	AEO20132.1	Linuron	Temp 22–30°C, *K*_m_ 5.8 μM, MW 55 KDa	*Variovorax* sp. SRS16	Agricultural soil, Denmark	Sørensen et al., 2005; Bers et al., 2012
HylA	Yctj-like family, metal-dependent hydrolase subfamily, amidohydrolase superfamily	AGF25452.1	OM	Temp 35°C, *K*_m_ 15.0 ± 0.8 μM (for linuron), MW 56 KDa	*Variovorax* sp. WDL1	Herbicide treatment soil, Belgium	Dejonghe et al., 2003; Bers et al., 2013
Phh	Metal-dependent hydrolase subfamily, amidohydrolase superfamily	ANU78861.1	DM and OM	pH 7.0–8.0, Temp 33–37°C, *K*_m_ 235.4 ± 37 μM (for diuron, different *K*_m_ calculated for all the substrates), MW 49.5 KDa	*Diaphorobacter* sp. LR2014-1	A disused pesticide factory, China	Zhang et al., 2018
Mhh	Amidase superfamily	ANU78862.1	Linuron and duron	pH 7.5, Temp 35°C, *K*_m_ 28.8 ± 1.7 μM, 21.5 ± 1.2 μM (for linuron and siduron), MW 50 KDa	*Diaphorobacter* sp. LR2014-1	A disused pesticide factory, China	Zhang et al., 2018
LahB	Metal-dependent hydrolase subfamily, amidohydrolase superfamily	QDQ16834.1	Linuron	pH 7.0, Temp 30°C, *K*_m_ 37.3 ± 1.2 μM (for linuron), MW 49.8 KDa	*Sphingobium* sp. SMB	Herbicide-contaminated soil, China	Zhang et al., 2020

*DM refers to *N*, *N*-dimethyl-substituted phenylurea herbicides; OM refers to *N*-methoxy-*N*-methyl-substituted phenylurea herbicides; MW refers to molecular weight.*

The first purified phenylurea hydrolase was obtained in 1971 from *Bacillus sphaericus*, which catalyzes the breakdown of *N-*methoxy*-N-*methyl phenylureas (OMs), but not DUR or other *N,N*-dimethyl phenylureas (DMs) (Engelhardt et al., 1971, 1973). Genetic characterization of the first DUR-degrading enzyme, PuhA, was performed by Turnbull et al. (2001). After this, PuhB (Khurana et al., 2009), LibA (Bers et al., 2012), HylA (Bers et al., 2013), Phh, Mhh (Zhang et al., 2018), and LahB (Zhang et al., 2020) were identified (Table 3). They can all transform PUHs into 3,4-DCA. PuhA and PuhB were identified in the DUR degraders *A. globiformis* D47 and *Mycobacterium brisbanense* JK1, respectively. In fact, despite both PuhA and PuhB being isolated from DUR-degrading bacteria, the catalytic efficiency of these two enzymes for linuron was relatively high, and they also hydrolyzed both DM and OM PUHs (Khurana et al., 2009). PuhA and PuhB were discovered in separate continents, which makes it challenging to elucidate the evolutionary origins of these enzymes. Horizontal gene transfer through genetic elements, such as plasmids and transposons, is a possible way to spread phenylurea-degrading genes. Dunon et al. (2013) reported that the number of insertion sequence IS1071 gene copies increased substantially after phenylurea treatment, indicating that horizontal gene transfer might occur in these degraders. However, Ozturk et al. (2020) analyzed the full-genome sequences of six *Variovorax* strains capable of degrading linuron, which were isolated from geographically distant locations. This result indicates that it is unlikely that they originated from a common ancestral linuron degrader, in contrast with the *s*-triazine degraders whose degradation genes are spread in a variety of bacterial populations (Wackett et al., 2002). LibA, which was identified in the linuron-degrading *Variovorax* sp. SRS16, has high specificity and only hydrolyzes linuron (Bers et al., 2011). Strain SRS16 was the first bacterium for which the genetic organization of the complete mineralization pathway of a PUH has been established. In SRS16, the enzymes dcaQTA_1_A_2_B (deoxygenation of DCA to chlorocatechol) and ccdCDEF (chlorocatechol ortho-cleavage) are involved in the transformation of dicholoroaniline to oxo-adipate. HylA was also identified in the *Variovorax* genus and could only hydrolyze *N*-methoxy-*N*-methyl-substituted phenylureas, including linuron, monolinuron, and metobromuron (Bers et al., 2013).

Recently, Zhang et al. (2018, 2020) identified the enzymes Phh and Mhh (from *Diaphorobacter* sp. LR2014-1) and LahB (*Sphingobium* sp. SMB). Phh and Mhh functioned in the complementary hydrolysis of PUHs, enabling strain LR2014-1 to hydrolyze both DM and OM PUHs (Zhang et al., 2018). Interestingly, Phh and Mhh are evolutionarily divergent. The new PUH-degrading enzyme LahB, which is similar to LibA, can only hydrolyze linuron and exhibits a narrow substrate spectrum (Zhang et al., 2020). Among these seven enzymes, HylA belongs to the YctJ-like family within the metal-dependent amidohydrolase superfamily. PuhA, PuhB, Phh, and LahB are members of this superfamily, whereas LibA and Mhh belong to the amidase superfamily (Khurana et al., 2009; Bers et al., 2012, 2013; Zhang et al., 2018, 2020).

Apart from these six hydrolases, recent studies have indicated that other novel DUR enzymes may exist in the environment. Coelho-Moreira et al. (2018) and Hu et al. (2020) found that the addition of cytochrome P450 inhibitors significantly reduced the ability of fungi to degrade DUR, suggesting that cytochrome P450 is an enzyme involved in the degradation of DUR, which is in agreement with a previous study developed by Abass et al. (2007), who considered cytochrome P450 to be an enzyme involved in demethylation of DUR in humans and other mammals. Moreover, exposure to DUR increases the production of lignin peroxidase (Coelho-Moreira et al., 2013; Mori et al., 2018). Coelho-Moreira et al. (2018) showed that the maximal production of laccase occurred simultaneously with maximal DUR degradation. They also confirmed that crude laccase extracts from the white-rot fungus *Ganoderma lucidum* could degrade. In contrast to Coelho-Moreira et al. (2018) and Henn et al. (2020) applied crude enzymatic extracts of *Pycnoporus sanguineus* but did not catalyze the degradation of DUR *in vitro*, despite the high degradation rate of *P. sanguineus* and the strong inductive effect of DUR on laccase synthesis and secretion. Hu et al. (2020) observed that DUR concentrations remained constant after incubating *T. versicolor* laccase in *in vitro* experiments, despite substantial laccase activity. Further studies are needed to elucidate the mechanisms of laccase, cytochrome P450, and other enzymes during DUR degradation.

## Preventive Bioremediation Using Diuron-Degrading Microorganisms

Bioaugmentation is considered an efficient way to remediate DUR-polluted sites by introducing specific degrading microorganisms (Cycoń et al., 2017; Arora et al., 2018; Feng et al., 2020; Lin et al., 2020). Although a considerable number of DUR-degrading microorganisms have been isolated, identified, and characterized and their metabolic mechanisms have been elucidated, their ability to remediate DUR-contaminated environmental matrices, such as water, soil, and subsurface material, remains a major challenge (Huang et al., 2017). Previous studies have focused on the degradation ability of bacteria and fungi in aqueous media (Ellegaard-Jensen et al., 2014; Villaverde et al., 2018). Recently, an increasing number of studies have focused on remediation in aquatic environments, slurry, and soil (Chen et al., 2014; Mishra et al., 2021). To prevent active ingredients from leaching into non-target areas, Carles et al. (2021) provided a preventive bioremediation method. Through the simultaneous application of the herbicide 2,4-D and degrading bacteria on planted mesocosms, they reported that 2,4-D was efficient in removing the weeds, and it was rapidly mineralized by the inoculated degraders.

The ability of microorganisms to remove xenobiotics is ultimately dependent on the bioavailability of herbicides and the surrounding conditions, including the soil type, the physicochemical properties of the soil, pH, humidity, temperature, nutrient availability, and oxygen level (Turnbull et al., 2001; Maqbool et al., 2016; Dias Guimaraes et al., 2018). Supplementation of soils with 1 and 2% biochar enhanced the adsorption, slowed desorption, and reduced the biodegradation of isoproturon (Sopena et al., 2012). Dias Guimaraes et al. (2018) reported that the chemical properties of the soil affect the DUR DT_50_ value, but no correlation between DUR degradation and organic carbon content and pH was found. The effect of soil chemical properties on adsorption may outweigh that of degradation. DUR adsorption in the soil is decreased with increasing pH values, with high adsorption levels at relatively low pH values (Liu et al., 2010). Higher cation exchange capacity values led to greater DUR colloid sorption, so lower herbicide degradation would be expected. A higher organic carbon content increased the number of degrading microbes but also increased the adsorption of DUR, making it unavailable for microbial degradation. Thus, the conditions must be optimized in the pollutants selected and their physicochemical properties (Grandclement et al., 2017).

Bioreactors are a novel way to treat effluents containing pesticide active ingredients *in situ* (La Cecilia and Maggi, 2017; Góngora-Echeverría et al., 2020). Recently, novel biobed bioremediation systems have been used to enhance the bioavailability of herbicides and create optimal conditions for herbicide biodegradation, achieving a decontamination efficiency of 75% for DUR (Delgado-Moreno et al., 2017). The oxygen level, nitrogen level, recycling ratios, and influent flow rates are generally considered to be strongly related to the efficiency of herbicide removal (Hu et al., 2020). According to Castañón-González et al. (2016), there is a higher degradation rate and less 3,4-DCA accumulation in an aerobic reactor than in an oxygen-limited reactor. However, Stasinakis et al. (2009) found that almost 60% of DUR was biodegraded when using 3,4-DCA as a major metabolite under aerobic conditions, whereas there was more than 95% degradation with DCPU as a major metabolite under anoxic conditions. Further studies are required to evaluate the removal efficiency in oxygen levels.

Survival, proliferation, immobilization, competition with indigenous bacteria, and catalytic capacity are important factors influencing the biodegradation ability of inoculants (Chen et al., 2013; Zhang et al., 2018, 2021; Bhatt et al., 2019). In addition to environmental factors, the characteristics of the microbes themselves also have an important effect on their degradation ability (Chan et al., 2021). Embedding functional enzymes and microbes with biomaterials or nanomaterials has been of increasing interest to facilitate *in situ* bioremediation. Organophosphorus hydrolase has been assembled in outer membrane vesicles to enhance the degradation of organophosphate pesticides, hydrolase recovery, and reuse capability (Su et al., 2017). Natural cellulose and herbivore waste are also suitable materials owing to their excellent biocompatibility. Liu et al. (2018) immobilized the strain *A. globiformis* D47 on the fiber networks of nanocellulose and showed that the application of bacteria-decorated nanocellulose produced a higher degradation rate of DUR under different conditions. Silkworm excrement has also been used to immobilize the same bacterium, showing a high survival rate and stable catalytic degradation of DUR (Liu et al., 2019).

## Conclusion and Future Perspectives

More recent attention has focused on the removal of DUR from contaminated sites. Bioremediation is one of the most effective and eco-friendly ways to degrade DUR. However, due to the poor culturability of microorganisms, only a few bacterial strains capable of mineralizing have been reported. Most strains can hydrolyze DUR to 3,4-DCA but fail to completely mineralize DUR. The molecule 3,4-DCA is a common product of the intermediate metabolism of DUR and linuron degradation; a combination of these degraders for co-metabolism degradation of coexisting herbicides could provide valuable insights for future research. DUR degraders do not seem to be limited to any specific genus or species, unlike in the PUH linuron, which has been reported to have the genus *Variovorax* as its predominant bacterial strain. Therefore, axenic culture or constructed consortia are needed to achieve the complete mineralization of DUR. The relationship and interaction of individuals in consortia should also be tested to optimize their performance.

A number of studies have evaluated the efficiency of biodegradation under *in vitro* conditions in liquid cultures. Laboratory studies on the degradation in soil or large-scale field studies *in situ* are also needed to integrate all the factors that could influence degradation and evaluate the chemistry, toxicity, and environmental fates of DUR and its metabolites. *In situ* removal of target pollutants, colonization, and immobilization are important for microorganisms to survive and degrade specific molecules. Compared with the direct utilization of degraders, embedding functional microbes in biomaterials or emerging nanomaterials will enhance their bioavailability and create optimal conditions to facilitate bioremediation in complex environments.

## Author Contributions

SC conceived of the presented idea. JL contributed to the writing and prepared the figures and tables. WZ, ZL, YH, PB, and SC participated in revising the manuscript. All the authors approved it for publication.

## Conflict of Interest

The authors declare that the research was conducted in the absence of any commercial or financial relationships that could be construed as a potential conflict of interest.

## Publisher’s Note

All claims expressed in this article are solely those of the authors and do not necessarily represent those of their affiliated organizations, or those of the publisher, the editors and the reviewers. Any product that may be evaluated in this article, or claim that may be made by its manufacturer, is not guaranteed or endorsed by the publisher.
